# Less Favorable Nutri-Score Consumption Ratings Are Prospectively Associated with Abdominal Obesity in Older Adults

**DOI:** 10.3390/nu16071020

**Published:** 2024-03-31

**Authors:** Jimena Rey-García, Diana María Mérida, Carolina Donat-Vargas, Helena Sandoval-Insausti, Montserrat Rodríguez-Ayala, José Ramón Banegas, Fernando Rodríguez-Artalejo, Pilar Guallar-Castillón

**Affiliations:** 1 Internal Medicine Department, Hospital Universitario Ramón y Cajal, Instituto Ramón y Cajal de Investigación Sanitaria, 28034 Madrid, Spain; jimena.reygarcia@gmail.com; 2 Department of Preventive Medicine and Public Health, School of Medicine, Universidad Autónoma de Madrid, 28029 Madrid, Spain; diana.merida@estudiante.uam.es (D.M.M.); ms.rodriguezayala@gmail.com (M.R.-A.); joseramon.banegas@uam.es (J.R.B.); fernando.artalejo@uam.es (F.R.-A.); 3 ISGlobal-Institut de Salut Global de Barcelona, 08036 Barcelona, Spain; cdonatvargas@gmail.com; 4 Unit of Cardiovascular and Nutritional Epidemiology, Institute of Environmental Medicine, Karolinska Institutet, 17177 Stockholm, Sweden; 5 Department of Nutrition, Harvard T.H. Chan School of Public Health, Boston, MA 02115, USA; helenagabar@gmail.com; 6 Department of Microbiology and Parasitology, Hospital Universitario La Paz, 28046 Madrid, Spain; 7 CIBERESP (CIBER of Epidemiology and Public Health), 28029 Madrid, Spain; 8 IMDEA-Food Institute, Campus de Excelencia Internacional (CEI)/Universidad Autónoma de Madrid (UAM) + Consejo Superior de Investigaciones Científicas (CSIC), 28049 Madrid, Spain

**Keywords:** abdominal obesity, Seniors-ENRICA 1 study, five-color Nutri-Score, metabolic risk, cohort study

## Abstract

Nutri-Score is a front-of-package (FOP) labeling designed to assist consumers in selecting healthier options at the point of purchase and ultimately enhance their health. This study aims to evaluate the association between the Nutri-Score system and incident abdominal obesity (AO) in community-dwelling older adults. A prospective cohort of 628 individuals aged ≥ 60 were recruited in Spain between 2008–2010 and were reexamined between 2015–2017. Dietary intake was evaluated utilizing a validated computerized dietary history. Food was categorized based on the Nutri-Score system into five levels from A (green, representing the best quality) to E (red, representing the poorest quality). A five-color Nutri-Score dietary index (5-CNS DI) in g/day/kg was calculated for each participant. AO was determined by a waist circumference (WC) of ≥102 cm for men and ≥88 cm for women. Logistic regression models were adjusted for the main potential confounders. During a mean six-year follow-up, 184 incident cases of AO occurred. The odds ratio (OR) and 95% confidence interval (CI) for AO, when comparing the highest and lowest quartiles of the 5-CNS DI, were 2.45 (1.17–5.14), with a *p*-value for trend of 0.035. In sensitivity analyses, the OR was 2.59 (1.22–5.52, *p*-trend: 0.032) after adjustment for WC at baseline, and 1.75 (0.74–4.18, *p*-trend: 0.316) after adjustment for ultra-processed food consumption. In conclusion, less favorable food-consumption ratings in the Nutri-Score are associated with incident AO in the elderly. These findings support the use of this FOP system to potentially improve metabolic health.

## 1. Introduction

Front-of-pack (FOP) nutrition labelling such as the Nutri-Score system aims to help consumers in selecting healthier food options at the point of purchase, with the goal being to enhance their overall health [1]. These are policy instruments that, when combined with educational initiatives, can promote healthy eating habits and mitigate the risk of diet-related chronic diseases [2]. FOP nutrition labels employ nutrient-profiling frameworks to evaluate the nutritional value of food products, displaying their health attributes in an accessible visual format [2]. Their purpose is to provide consumers with nutritional information that is both easy to comprehend and read, while also motivating the food manufacturing industry to enhance the nutritional quality of their products [3].

A recent systematic review of the relevant literature suggests that FOP nutrition labelling leads to significant improvements in food choices [4]. Specifically, the Nutri-Score system enhanced consumers’ capability to accurately assess food based on its nutritional value, encouraged their decision-making in selecting healthier options, and augmented their proficiency in selecting suitable portion sizes [5]. 

Furthermore, in several population-based cohorts, a less favorable Nutri-Score rating food consumption has been prospectively associated with a higher risk of chronic diseases [5]. This association has been shown for cardiovascular disease [6], metabolic syndrome [7], decline in renal function [8], incident frailty [9], cancer [10,11], as well as all-cause mortality [12].

Abdominal obesity (AO) is a well-known risk factor for cardiometabolic disorders. In older adults specifically, AO is a risk factor for cardiometabolic disease independent of general obesity [13]. In this population, AO is also associated with increased risk of disability [14] and sarcopenic obesity [15].

Hence, this study’s objective was to conduct a prospective evaluation of the association between food intake categorized according to the Nutri-Score system, and the onset of abdominal obesity (AO) in older adults residing in the community, while thoroughly adjusting for an extensive range of potential confounding factors.

## 2. Materials and Methods

### 2.1. Research Methodology and Participant Selection

Data were obtained from the Seniors-ENRICA 1 cohort, the methodology of which has been previously documented [16]. This cohort was established in Spain between 2008–2010 with 3521 community-based individuals aged ≥ 60 years. Data were gathered by qualified staff in three consecutive stages. At baseline, a telephone interview was conducted to obtain information on sociodemographic factors, lifestyle, and morbidity. Subsequently, two home visits were conducted to gather urine and blood specimens, assess anthropometric variables and blood pressure, and collect dietary information. Participants were followed-up with until 31 December 2015, achieving an average follow-up duration of six years, at which point data collection was updated including a physical examination.

**Figure 1:**
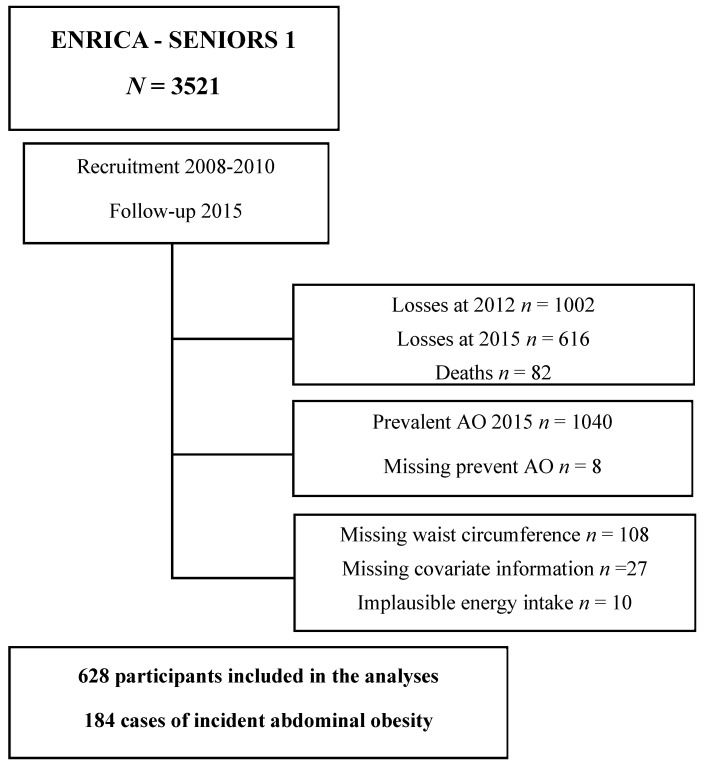
Participant flowchart.

Within the cohort of 3521 participants, 1618 were excluded due to attrition during the follow-up periods in 2012 and 2015, with 1002 and 616 participants lost in each year, respectively. During follow-up, 82 deaths occurred. Of the remaining 1821 participants, 1040 were excluded because they had prevalent AO at baseline, and 8 because of missing data on waist circumference at baseline. We also excluded 10 participants with implausible energy intake (<600 or >4200 Kcal/day for men, or <400 or >3500 Kcal/day for women), and 27 who lacked data on other covariables (weight, body mass index (BMI), hypertension, diabetes, food consumption, and hours of TV viewing). Thus, the final analyses were carried out on 628 participants, among which 184 developed incident abdominal obesity (AO) during the follow-up period (Figure 1).

The Clinical Research Ethics Committee of the University Hospital of La Paz in Madrid, Spain approved the baseline and follow-up studies. All participants provided written informed consent.

### 2.2. Research Variables

#### 2.2.1. Dietary Intake Evaluation and Calculation of the Nutri-Score

Dietary intake over the past year was evaluated through a validated computerized dietary history tool (DH-ENRICA) that was administered by skilled interviewers. This questionnaire gathers uniform data on 880 food items and 184 recipes for commonly consumed dishes in Spain. A collection of 129 photographs were used to estimate food portion sizes [17]. Consumption was considered habitual if a food was consumed at least once every two weeks. Intakes of energy and nutrients were determined utilizing standard Spanish food composition databases [18,19]. 

The Nutri-Score is a FOP labelling system that classifies products according to their nutritional value [20,21]. In our study, this labelling system was applied to each food product suitable for packaging and consumed by the study participants using the updated algorithm of 2022 for food and 2023 for beverages [22,23]. A score was assigned based on the nutrient content of the packaged food. Fresh produce (predominantly fruits and vegetables) and unprocessed meat and fish were omitted from the calculation of the Nutri-Score. For each packaged food item consumed, positive points were assigned ranging from 0 to 10 based on its content of four specific elements: total energy (kj), sugars (g), saturated fats (g), and sodium (mg). Conversely, foods were allocated negative points, varying from 0 to −5, depending on their amounts of five beneficial components: proportion of fruits, vegetables, legumes, nuts, and healthy oils, as well as dietary fiber (g), and protein (g). Consequently, a continuous Nutri-Score from −15 to +40 was determined for each consumed food item, where a higher score indicates a lower nutritional quality. This continuous score was segmented into five categories, resulting in a five-color Nutri-Score (5-CNS) assigned to each food item. Subsequently, foods were allocated into one of five classifications, represented by letters (A, B, C, D, and E) and associated colors, ranging from dark green for letter A, denoting superior nutritional quality, through to light green (B), yellow (C), orange (D), and dark orange (E) for the lowest nutritional quality [21].

For each participant, we calculated the following four dietary indexes (DI) based on their food consumption and the Nutri-Score previously assigned to each food:

1. The five-color Nutri-Score dietary index (5-CNS DI, in grams per day per kilogram): This index was determined by totaling the consumed quantities of each packaged food and beverage (grams per day), each multiplied by its respective 5-CNS rating (where A is scored as 1 and E as 5), and then dividing this total by the individual’s body weight in kilograms. In the present analysis this DI was considered the main exposure.
2. The continuous Nutri-Score dietary index (in grams per day per kilogram): This index was computed by summing the consumption amounts of all packaged foods and beverages (grams per day), each amount multiplied by its respective continuous Nutri-Score value (which varies from −15 to +40). This sum was divided by the individual’s body weight in kilograms.
3. The five-color Nutri-Score dietary index (5-CNS DI as a percentage of energy intake): This index was computed using the 5-CNS DI, where food consumption is represented as a percentage of total energy intake (percent energy per kilogram).
4. The continuous Nutri-Score dietary index (as a percentage of energy intake): This index was derived by calculating the continuous Nutri-Score DI, where food consumption is quantified as a percentage of total energy intake (percent energy per kilogram).

#### 2.2.2. Abdominal Obesity

Waist circumference measurements were taken at baseline (2008–2010) and, upon conclusion of the follow-up phase (in 2015), through in-home physical assessments conducted by qualified staff. Incident AO was calculated according to the World Health Organization recommended cut-off points as waist circumference ≥ 102 cm in men and ≥88 cm in women in 2015 [24].

#### 2.2.3. Other Variables

At the beginning of the study, sociodemographic, lifestyle, and morbidity data were collected. Weight and height measurements were conducted at participants’ homes under standardized conditions, and BMI was calculated. Self-reported data were collected regarding sex, age, educational level, smoking habits, former drinker status, time spent watching TV, and leisure time physical activity [16]. Hypertension was defined as blood pressure ≥ 140 and/or 90 mmHg, or use of antihypertensive medication. Participants in the study also provided information on physician-diagnosed conditions, including chronic respiratory disease, coronary heart disease, stroke, osteoarthritis/arthritis, cancer, depression requiring treatment, and diabetes. Participants disclosed all medications they were consuming, which were verified by a nurse against the actual medicine packages kept at the participants’ homes. Finally, the Mediterranean Diet Adherence Score (MEDAS), a 14-item validated tool designed to measure the adherence to the Mediterranean diet which methods are reported elsewhere, was assessed [25]. Ultra-processed food (UPF) was categorized based on the NOVA classification system [26].

### 2.3. Statistical Analysis

Based on the four derived Nutri-Score DIs, participants were classified into sex-specific quartiles. To assess the risk of abdominal obesity within these sex-specific quartiles for each Nutri-Score DI, logistic regression analysis was conducted and odds ratios (OR) along with their respective 95% confidence intervals (CI) were determined. The *p*-value for linear trends was calculated by using the quartiles of each DI as a continuous variable. Three logistic regression models were constructed, incorporating adjustments for potential confounding variables. Model 1 was adjusted for sex, age, educational level (primary or less, secondary, and university), and total energy intake (kcal/d). Model 2 was additionally adjusted for smoking (current, former, and never smoker), physical activity at leisure time (Mets/h/week), time watching TV (h/week), total sleep time (minutes/day), total ethanol consumption (g/day), BMI (continuous), chronic obstructive pulmonary disease/asthma, coronary heart disease, hypercholesterolemia, hypertension, diabetes, cancer (yes/no), arthrosis (yes/no), arthritis (yes/no), and number of medications (0, 1–3, >3). Model 3 was further adjusted for the MEDAS without wine (0–13 points). Sensitivity analyses were performed by adjusting for waist circumference (cm) at baseline and for UPF consumption (classified as NOVA-4).

To assess the dose–response relationship, restricted cubic-splines with three knots (10th, 50th, and 90th percentile) were fitted, showing the ORs for the association of AO throughout the continuous Nutri-Score DI in g/day/kg as well as their 95% CI. Data analysis was conducted utilizing STATA/SE version 14.1, developed by StataCorp in College Station, TX, USA. Statistical significance was established at a two-sided *p*-value of less than 0.05.

## 3. Results

Of the 628 participants included in the analyses (44.9% women; mean (SD) 6.02 ± 5.76 years), 184 developed AO after a mean six-year follow-up. The mean (SD) for the 5-CNS DI in g/day/kg at baseline was 33.5 (12.7). In the lowest quartile (best nutritional quality) of the 5-CNS DI, the consumption of foods labelled A was 146 (108) grams per day and 39 (35) grams per day for foods labelled E. Within the highest quartile (poorer nutritional quality), the amounts were 161 (91) and 164 (152) grams per day, respectively. Participants in the highest quartile of the 5-CNS DI per day per kilogram exhibited higher energy consumption and reduced intake of fresh foods, presented with a lower BMI, and showed a lower likelihood of hypertension and medication use. However, they were more inclined to have been former drinkers and demonstrated increased consumption of UPF compared to those in the lowest quartile (Table 1). 

In the fully adjusted model, OR (95% CI) for AO across the increasing quartiles of the 5-CNS Dietary Index (DI) per gram per day per kilogram were as follows: 1 (reference), 1.29 (0.72–2.32), 1.25 (0.66–2.36), and 2.45 (1.17–5.14); with a *p*-value for trend at 0.035. The risk of AO increased by 24% (0–53%) with a 10-unit increase in this DI. When comparing the highest to the lowest quartiles of the continuous Nutri-Score DI in g/day/kg, the OR (95% CI) for AO was 2.52 (1.22–5.20), *p*-trend = 0.023, with an 8% (0–17%) increased risk of AO per 10-unit increase in this DI (Table 2). 

Comparable results were observed for the two DIs based on the percentage of energy; the respective values stood at 2.03 (0.98–4.23), *p*-trend = 0.024, for the 5-CNS DI, and 2.24 (1.07–4.68), *p*-trend = 0.031, for the continuous Nutri-Score DI (Table 3).

**Figure 2:**
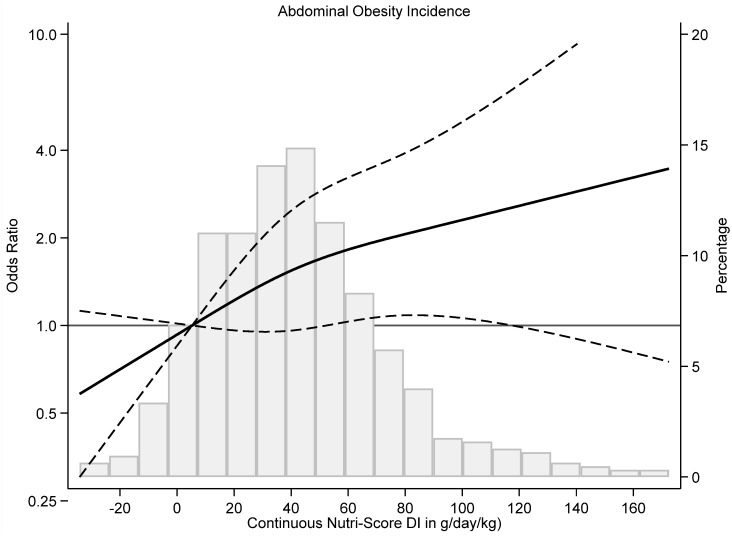
Restricted-cubic splines of association of continuous Nutri-Score DI in g/day/kg with risk of abdominal obesity in Seniors-ENRICA 1 study (from 2008–2010 and 2015). Analyses were adjusted as in Model 3 in Table 2 [adjustment for sex, age (continuous), total energy intake (kcal/d), and educational level (primary or less, secondary, and university), smoking (current, former, and never smoker former drinker status (yes/no), physical activity at leisure time (METS/h/week), time watching TV (h/week), total sleep time (minutes/day), total ethanol consumption (grams/day), body mass index (continuous), chronic obstructive pulmonary disease/asthma (yes/no), coronary heart disease (yes/no), hypercholesterolemia (yes/no), hypertension (yes/no), diabetes (yes/no), cancer (yes/no), arthrosis (yes/no), arthritis (yes/no), number of medications (0, 1–3, >3). MEDAS index score excluding wine (0–13 points)].

When the dose–response was assessed using the continuous Nutri-Score DI, a positive linear relationship was observed which indicated that the higher the index, the higher the incidence of AO (Figure 2).

In the sensitivity analyses, the OR (95% CI) for AO after adjusting for waist circumference at baseline when comparing extreme quartiles of the 5-CNS DI was 2.59 (1.22–5.52); *p*-trend = 0.032. In addition, the correlation between the 5-CNS DI and UPF consumption was 0.68. The OR (95% CI) after adjustment for UPF consumption at baseline was: 1.75 (0.74–4.18); *p*-trend = 0.316 (Table S1). Comparable results were obtained for the continuous Nutri-Score DI in g/day/kg and in the percentage of energy (Table S2). 

## 4. Discussion

In this cohort of community-dwelling older adults from Spain, individuals following a diet with a less favorable Nutri-Score exhibited approximately a 24% higher risk of abdominal obesity (AO) for every 10-unit increase in the 5-CNS DI (g/day/kg). This association persisted after adjustment for BMI as well as adherence to the Mediterranean diet. The findings indicate that a diet of lower nutritional quality, evaluated using the Nutri-Score system, is significantly associated to a higher incidence of abdominal obesity in the elderly population.

Our findings align with current research on the topic. The influence of food’s nutritional quality (levels of saturated fat, salt, calories, sugar, etc.) is widely recognized [27]. Several epidemiological and interventional studies have shown the association between the quality of the food consumed and various diseases. Regarding the individual components of the Nutri-Score, the higher incidence and prevalence of obesity was associated with higher consumption of energy [28], sugar [29], saturated fat [30], and sodium [31], but a protective association was found with higher consumption of fruits and vegetables [32], fiber [33], and protein [34]. Indeed, FOP such as the Nutri-Score intend to be a synthesis of these evidences and, as such, several prospective cohorts (i.e., SU. VI.MAX, the NutriNet-Santé, the EPIC, and the Seniors-ENRICA 1 cohort), have shown that a less favorable Nutri-Score is associated with numerous diseases [6,7,8,9,10,11] and all-cause mortality [12]. 

In addition to the association derived from each nutrient component of the Nutri-Score (part of the algorithm for its calculation) which has been widely studied, another possible underlying mechanism of this association may be related to the degree of processing of the foods consumed. Studies have shown that about 84% of foods labelled D or E in the Nutri-Score system are highly processed or UPF [35]. A recent systematic review of prospective studies showed a link between the consumption of UPF and a higher incidence of obesity, along with an elevated risk of cardiometabolic conditions [36]. In a study from Sandoval-Insausti et al. [37], an association between UPF consumption and incident AO was identified. These findings have been reproduced in other worldwide cohorts such as the Brazilian Longitudinal Study of Adult Health (ELSA-Brasil) cohort [38], the Ribeirão Preto cohort [39], the NutriNet-Santé cohort [40], the University of Navarra Follow-Up (SUN) cohort study [41], the China Health and Nutrition Survey (CHNS) [42], the EPIC [43], and the National Diet and Nutrition Survey Rolling Program (NDNS) in the United Kingdom [44]. All showed a positive association between UPF consumption and the development of AO.

Biological mechanisms have been proposed to link the degree of food processing with AO beyond its nutritional quality including the content of additives added to these products. Additives have been associated with changes in the gut microbiota’s composition promoting inflammatory diseases [45], with potentially important implications for body weight and adiposity [46]. Additionally, UPFs are generally packaged in plastics, and numerous plasticizers (such as bisphenol A) have been shown to be associated with obesity, possibly acting as endocrine disruptors [47].

In our analyses, 48.3% of the association between 5-CNS DI and AO was explained by UPF consumption, and the remainder could be explained by the Nutri-Score components themselves (energy, sugar, saturated fat, sodium, fruits and vegetables, fiber, and protein content). Therefore, the extent of food processing and the nutritional quality, as denoted by the Nutri-Score, are interconnected, representing complementary aspects (since UPF tends to have a higher score in the 5-CNS DI and at the same time tends to have a lower nutritional quality) [27]. Considering these two dimensions together, our results make it possible to improve dietary advice by promoting the consumption of products with a better Nutri-Score, which generally tend to be not only those with a better nutritional quality, but also those with the lowest level of processing.

Public health strategies are essential to guide consumer choices and achieve a substantial population impact on preventing cardiovascular disease through diet [6]. Among FOP, the graphical design of Nutri-Score facilitates nutritional literacy, thus favoring consumer choice [1,48,49,50]. Nevertheless, there is also evidence to suggest that the Nutri-Score system may require more public education compared to other intuitive symbols (such as warning labels) [51]. For example, it is often thought that Nutri-Score ‘punishes’ some healthy products such as olive oil, previously categorized as C. Nevertheless, Nutri-Score is intended to make it simpler to compare foods within the same category. If we compare different types of oil, olive oil gets the best score. This concept requires people to understand how to interpret the Nutri-Score properly.

Another aspect that encourages the use of FOPs is the pressure on companies to adapt and improve the nutritional quality of their products. However, there is limited evidence on the impact of FOP nutrition labelling on food reformulation [52]. The impact of such policies on reformulation is likely to be greater if they are mandatory, coordinated with other regulations, and thoroughly monitored and evaluated [52]. In the European Union, the use of FOPs is recommended but not mandatory. This may be the reason why the impact of FOPs on reformulation may be limited (achieving only minor changes). In addition, policies targeting reformulation and wider food system policies will be needed to significantly improve diets.

The relationship between dietary patterns guided by the Nutri-Score system and abdominal obesity holds significant social relevance. First, the Nutri-Score is practical and easy-to-understand, making it accessible and useful for individuals with lower levels of education and older adults. The Nutri-Score system also enhances consumer empowerment by boosting their capacity to make well-informed decisions. The prevalence of AO among older adults in Spain is high and very difficult to reverse. It is estimated that 61.6% of older adults in Spain already have AO, with this prevalence being even higher among women (69.7%) [53]. AO is also associated with an increased risk of disability [14], and an increase in fat mass may be associated with sarcopenic obesity [15]. Several epidemiological studies suggest that this syndrome is associated with accelerated functional decline, increased risk of multiple diseases and, ultimately, increased mortality [15]. Therefore, any effort to reduce the incidence of AO may be beneficial.

The study also presents certain limitations. First, the number of cases of incident AO was relatively low due to the high prevalence of AO at baseline. However, the association attained was statistically significance. Second, repeated dietary measurements were not considered, and diet may have changed since baseline. Third, because diet was self-reported, recall bias cannot completely be ruled out. Fourth, despite comprehensive adjustments in the statistical analyses for numerous potential confounding factors, the possibility of some residual confounding cannot be entirely excluded. Our study also has several strengths. First, prospective design makes it more likely that the time order of the association will be examined. Second, dietary intake was evaluated using a validated dietary history that was gathered by trained personnel. Third, the findings remained robust following adjustments for numerous potential confounders.

## 5. Conclusions

A diet characterized by a less favorable Nutri-Score rating is prospectively associated with an increased risk of developing AO. Our findings endorse the adoption of the Nutri-Score front-of-pack (FOP) labeling system as an effective public health measure to improve dietary nutritional quality.

## Figures and Tables

**Table 1:** Baseline characteristics of cohort participants according to sex-specific quartiles of five-color Nutri-Score dietary index (5-CNS DI) in g/day/kg (*n* = 628).

Five-Color Nutri-Score (5-CNS DI) in g/Day/kg						
		Q1 (Best Diet Quality)	Q2	Q3	Q4 (Worse Diet Quality)	*p* for Linear Trend
*n*		158	162	162	146	
5-CNS DI in g/day/kg, mean ± SD		20.2 ± 4.10	28.8 ± 24.43	35.7 ± 2.39	50.6 ± 12.2	<0.001
Continuous Nutri-Score DI in g/day/kg, mean ± SD		20.3 ± 20.09	31.9 ± 21.0	46.1 ± 24.1	76.3 ± 42.0	<0.001
5-CNS DI based on the % of energy, mean ± SD		1.76 ± 0.44	2.08 ± 0.33	2.24 ± 0.38	2.45 ± 0.40	<0.001
ontinuous Nutri-Score DI based on the % of energy, mean ± SD		3.72 ± 2.48	4.55± 2.03	5.36 ± 2.29	6.10 ± 2.57	<0.001
Packaged foods (g/d), mean ± SD						
	Label A	146 ± 108	155 ± 92.8	167 ± 95.7	161 ± 91.1	0.092
	Label B	223 ± 108	289 ± 123	315 ± 146	362 ± 202	<0.001
	Label C	144 ± 83.2	230 ± 98.9	290 ± 120	375 ± 207	<0.001
	Label D	45.7 ± 44.4	59.4 ± 53.5	75.2 ± 61.8	116 ± 106	<0.001
	Label E	38.9 ± 35.4	59.8 ± 43.5	89.4 ± 65.0	164 ± 152	<0.001
Energy (Kcal), mean ± SD		1663 ± 410	1945 ± 450	2141 ± 458	2373 ± 470	<0.001
Sex (women), %		44.3	45.1	44.4	45.9	0.823
Age, mean ± SD		67.3 ± 5.80	66.6 ± 5.37	66.9 ± 5.31	67.3 ± 6.56	0.886
Educational level (%)						0.714 ^†^
	Primary or less	36.7	41.4	41.4	41.1	
	Secondary	29.8	29.0	28.4	34.3	
	University	33.5	29.6	30.3	24.7	
Smoking, %						0.700 ^†^
	Current smoker	14.6	14.8	11.7	16.4	
	Former smoker	33.5	26.5	28.4	28.8	
	Never smoker	51.9	58.6	59.9	54.8	
Former drinker status, %		6.33	7.41	4.32	13.01	0.094
Physical activity at leisure time (Mets/h/week), mean ± SD		24.7 ± 15.7	25.8 ± 17.4	22.9 ± 14.4	24.3 ± 16.7	0.453
Time watching TV (h/week), mean ± SD		15.4± 9.46	15.3 ± 8.93	14.6 ± 10.6	15.7 ± 8.47	0.956
Total sleep time (minutes/day), mean ± SD		428 ± 85.8	439 ± 81.4	434 ± 81.0	435 ± 77.1	0.578
Body Mass Index (kg/m^2^), mean ± SD		26.2 ± 2.44	25.7 ± 2.43	25.6± 2.49	24.9 ± 2.82	<0.001
Waist circumference (cm), mean ± SD		89.4 ± 8.69	88.3 ± 9.12	88.9 ± 9.0	86.7 ± 9.85	0.026
Ethanol consumption (g/day), mean ± SD		10.6 ± 16.4	10.7 ± 17.9	12.9 ± 18.2	9.12 ± 15.0	0.781
Coronary heart disease, %		0.00	2.47	0.00	0.68	0.885
Chronic respiratory disease, %		3.16	6.17	4.94	5.48	0.465
Hypertension, %		60.1	61.7	61.1	53.4	0.432 ^†^
Diabetes, %		10.8	10.49	6.17	5.48	4.779 ^†^
Cancer, %		1.90	1.23	3.09	1.37	0.928
Osteoarthritis, %		34.8	35.8	35.2	37.0	0.739
Arthritis, %		5.70	7.41	8.64	9.59	0.183
Number of medications, %						0.751 ^†^
	0	33.5	37.0	38.3	41.1	
	1–3	55.7	49.4	51.9	47.3	
	>3	10.8	13.6	9.88	11.6	
MEDAS Score (excluding wine), mean ± SD		7.77 ± 1.65	7.48 ± 1.68	7.17 ± 1.57	6.61 ± 1.72	<0.001
Ultra-processed food (g/day)		116 ± 80	180 ± 98	245 ± 119	400 ± 223	<0.001

SD: standard deviation; 5-CNS: five-color Nutri-Score; DI: dietary index; ^†^ chi-squared test. Categories of the five-color Nutri-Score (5-CNS DI) in g/day/kg: Q1 (9.1523.95); Q2: (24.00–31.12); Q3 (38.98–31.23); Q4 (39.13–96.43) in men and Q1 (11.80–26.70); Q2: (26.84–33.56); Q3 (33.57–41.13); Q4 (41.21–120.49) in women.

**Table 2:** Risk of abdominal obesity over a six-year follow-up according to the Nutri-Score dietary indexes in g/day/kg (*n* = 628).

Five-Color Nutri-Score (5-CNS DI) in g/Day/kg						
	Sex-Specific Quartiles of the 5-CNS DI in g/day/kg					
	Q1 (Best Diet Quality)	Q2	Q3	Q4 (Worse Diet Quality)	*p*-Trend	Per 10 Unit-Increment
Interquartile range (g/day/kg)	17.78–23.46	27.04–30.46	34.13–37.62	42.94–54.78		
Cases/*n*	47/158	44/162	39/162	41/146		
Model 1, OR (95% CI)	1 (Ref.)	0.94 (0.56–1.56)	0.83 (0.48–1.44)	1.08 (0.59–1.99)	0.923	0.98 (0.82–1.17)
Model 2, OR (95% CI)	1 (Ref.)	1.34 (0.75–2.39)	1.32 (0.70–2.46)	2.71 (1.33–5.50)	0.012	1.28 (1.04–1.57)
Model 3, OR (95% CI)	1 (Ref.)	1.29 (0.72–2.32)	1.25 (0.66–2.36)	2.45 (1.17–5.14)	0.035	1.24 (1.00–1.53)
**Continuous Nutri-Score DI in g/day/kg**						
	**Sex-Specific Quartiles of the Continuous Nutri-Score DI in g/day/kg**					
	**Q1** **(Best Diet Quality)**	**Q2**	**Q3**	**Q4** **(Worse Diet Quality)**	***p*** **-Trend**	**Per 10-Unit Increment**
Interquartile range (g/day/kg)	1.16–14.32	26.52–35.75	45.12–54.07	69.01–100.82		
Cases/*n*	38/161	45/160	41/159	47/148		
Model 1, OR (95% CI)	1 (Ref.)	1.43 (0.85–2.40)	1.36 (0.79–2.33)	2.14 (1.19–3.87)	0.022	1.05 (0.99–1.11)
Model 2, OR (95% CI)	1 (Ref.)	1.62 (0.90–2.89)	1.59 (0.87–2.92)	2.73 (1.41–5.31)	0.006	1.09 (1.02–1.17)
Model 3, OR (95% CI)	1 (Ref.)	1.58 (0.87–2.84)	1.52 (0.81–2.85)	2.52 (1.22–5.20)	0.023	1.08 (1.00–1.17)

5-CNS: five-color Nutri-Score, DI: dietary index, OR: odds ratio, CI: confidence intervals. Model 1 was adjusted for sex, age (continuous), total energy intake (kcal/d), and educational level (primary or less, secondary, and university); Model 2 was adjusted for smoking (current, former, and never smoker), former drinker status (yes/no), physical activity at leisure time (METS/h/week), time watching TV (h/week), total sleep time (minutes/day), total ethanol consumption (grams/day), body mass index (continuous), chronic obstructive pulmonary disease/asthma (yes/no), coronary heart disease (yes/no), hypercholesterolemia (yes/no), hypertension (yes/no), diabetes (yes/no), cancer (yes/no), arthrosis (yes/no), arthritis (yes/no), and number of medications (0, 1–3, >3). Model 3 was further adjusted by MEDAS index score excluding wine (0–13 points).

**Table 3:** Risk of abdominal obesity over a six-year follow-up according to Nutri-Score dietary indexes based on percentage of energy (*n* = 628).

Five Color Nutri-Score DI (5-CNS DI) Based on the Percentage of Energy						
	Sex-Specific Quartiles of the 5-CNS DI Based on the % of Energy					
	Q1 (Best Diet Quality)	Q2	Q3	Q4 (Worse Diet Quality)	*p*-Trend	Per 1-Unit Increment
Interquartile range (% of energy)	1.44–1.74	1.93–2.06	2.20–2.32	2.51–2.87		
Cases/*n*	35/157	41/158	51/157	44/156		
Model 1, OR (95% CI)	1 (Ref.)	1.33 (0.78–2.26)	1.86 (1.10–3.12)	1.60 (0.92–2.78)	0.045	1.48 (0.98–2.24)
Model 2, OR (95% CI)	1 (Ref.)	1.41 (0.78–2.56)	2.29 (1.26–4.14)	2.19 (1.16–4.14)	0.005	1.88 (1.15–3.07)
Model 3, OR (95% CI)	1 (Ref.)	1.37 (0.74–2.53)	2.19 (1.17–4.11)	2.03 (0.98–4.23)	0.024	1.76 (0.98–3.15)
**Continuous Nutri-Score DI Based on the % of Energy**						
	**Sex-Specific Quartiles of the Continuous Nutri-Score DI Based on the % of Energy**					
	**Q1** **(Best Diet Quality)**	**Q2**	**Q3**	**Q4** **(Worse Diet Quality)**	***p*** **-Trend**	**Per 1-Unit Increment**
Interquartile range (% of energy)	1.58–2.74	3.66–4.367	5.08–5.84	6.95–8.95		
Cases/*n*	31/160	45/157	46/158	49/153		
Model 1, OR (95% CI)	1 (Ref.)	1.77 (1.0–3.0)	1.90 (1.11–3.25)	2.27 (1.32–3.93)	0.004	1.08 (1.00–1.16)
Model 2, OR (95% CI)	1 (Ref.)	1.74 (0.96–3.17)	2.15 (1.18–3.92)	2.34 (1.25–4.35)	0.007	1.08 (0.99–1.17)
Model 3, OR (95% CI)	1 (Ref.)	1.72 (0.92–3.19)	2.09 (1.10–4.00)	2.24 (1.07–4.68)	0.031	1.05 (0.95–1.17)

5-CNS: five-color Nutri-Score, DI: dietary index, OR: odds ratio, CI: confidence intervals. Model 1 was adjusted for sex, age (continuous), total energy intake (kcal/d), and educational level (primary or less, secondary, and university); Model 2 was adjusted for smoking (current, former, and never smoker), former drinker status (yes/no), physical activity at leisure time (METS/h/week), time watching TV (h/week), total sleep time (minutes/day), total ethanol consumption (grams/day), body mass index (continuous), chronic obstructive pulmonary disease/asthma (yes/no), coronary heart disease (yes/no), hypercholesterolemia (yes/no), hypertension (yes/no), diabetes (yes/no), cancer (yes/no), arthrosis (yes/no), arthritis (yes/no), and number of medications (0, 1–3, >3). Model 3 was further adjusted by MEDAS index score excluding wine (0–13 points).

## Data Availability

Data is contained within the article.

## Supplementary Materials

The following supporting information can be downloaded at: https://www.mdpi.com/article/10.3390/nu16071020/s1, Table S1. Sensitivity analysis for the association between the Nutri-Score Dietary Indexes in g/day/kg and the risk of abdominal obesity (N = 628). Table S2. Sensitivity analysis for the association between the Nutri-Score Dietary Indexes based on the percentage of energy and the risk of abdominal obesity (N = 628).

## Abbreviations

5-CNS DI	Five-Color Nutri-Score Dietary Index
BMI	Body mass index
CHNS	China Health and Nutrition Survey study and the National Diet
CI	Confidence intervals
DH-ENRICA	validated computer-based dietary history from the Study on Nutrition and Cardiovascular risk factors in Spain
DI	Dietary Index
ELSA-Brasil cohort	The Brazilian Longitudinal Study of Adult Health, cohort
ENRICA	Study on Nutrition and Cardiovascular risk factors in Spain
EPIC	European Prospective Investigation into Cancer and Nutrition cohort
FOP	Front-of-packages
NDNS	Nutrition Survey Rolling Programme
MEDAS	Mediterranean Diet Adherence Score
OR	Odds Ratio
SD	Standard Deviation
Seniors-ENRICA Cohort	Seniors Study on Nutrition and Cardiovascular risk factors in Spain cohort
SU.VI.MAX	The Supplementation en Vitamines et Mineraux Antioxydants cohort
SUN	University of Navarra Follow-Up cohort study
UPF	Ultra-processed food
