# Supplementary material for: Less Favorable Nutri-Score Consumption Ratings Are Prospectively Associated with Abdominal Obesity in Older Adults
Source: Nutrients. 2024 Mar 31;16(7):1020. doi: 10.3390/nu16071020 (PMC11013145; doi:10.3390/nu16071020)
Supplement: Supplementary file 1 [file nutrients-16-01020-s001.zip › nutrients-2884052-supplementary.pdf]

Less Favorable Nutri-Score Consumption Ratings are Associated with Abdominal Obesity in older adults: A Prospective Cohort Study

Table S1. Sensitivity analysis for the association between the Nutri-Score Dietary Indexes in g/day/kg and the risk of abdominal obesity (N=628).

Five-Color Nutri-Score (5-CNS DI) in g/day/kg

	Sex-specific quartiles of the 5-CNS DI in g/day/kg					
	**Q1 (best diet quality)**	**Q2**	**Q3**	**Q4 (worse diet quality)**	**P-trend**	**Per 10 unit-increment**
Interquartile range (g/day/kg)	17.78-23.46	27.04-30.46	34.13-37.62	42.94-54.78		
Cases/n	47/158	44/162	39/162	41/146		
Model 4, OR (95% CI)	1 (Ref.)	1.40 (076-2.56)	1.26 (0.66-2.43)	2.59 (1.22-5.52)	0.032	1.26 (1.02-1.57)
Model 5, OR (95% CI)	1 (Ref.)	1.15 (0.62-2.11)	1.01 (0.50-2.03)	1.75 (0.74-4.18)	0.316	1.12 (0.88-1.43)

Continuous Nutri-Score DI in g/day/kg

	Sex-specific quartiles of the Continuous Nutri-Score DI in g/day/kg					
	**Q1 (best diet quality)**	**Q2**	**Q3**	**Q4 (worse diet quality)**	**P-trend**	**Per 10-unit increment**
Interquartile range (g/day/kg)	1.16-14.32	26.52-35.75	45.12-54.07	69.01-100.82		
Cases/n	38/161	45/160	41/159	47/148		
Model 4, OR (95% CI)	1 (Ref.)	1.61 (0.8-2.94)	1.70 (0.90-3.22)	2.69 (1.29-5.61)	0.013	1.09 (1.01-1.18)
Model 5, OR (95% CI)	1 (Ref.)	1.45 (0.79-2.64)	1.32 (0.69-2.53)	2.03 (0.95-4.36)	0.116	1.05 (0.97-1.14)

5-CNS: Five-Color Nutri-Score, DI: dietary index, OR: Odds Ratio, CI: Confidence Intervals.

Model 4 was adjusted as in Model 3 [for sex, age (continuous), total energy intake (kcal/d) and educational level (primary or less, secondary, and university), smoking (current, former, and never smoker former drinker status (yes/no), physical activity at leisure time (METS/h/week), time watching TV (h/week), total sleep time (minutes/day), total ethanol consumption (grams/day), body mass index (continuous), chronic obstructive pulmonary disease/asthma (yes/no), coronary heart disease (yes/no), hypercholesterolemia (yes/no), hypertension (yes/no), diabetes (yes/no), cancer (yes/no), arthrosis (yes/no), arthritis (yes/no), number of medications (0, 1 to 3, and >3), the MEDAS Index Score excluding wine (0-13 points)] with further adjustment for waist circumference at baseline (cm); and **Model 5** was adjusted as in model 3 with further adjustment for ultra-processed food consumption (sex-specific quartiles).

Table S2. Sensitivity analysis for the association between the Nutri-Score Dietary Indexes based on the percentage of energy and the risk of abdominal obesity (N=628).

Five Color Nutri-Score DI (5-CNS DI) based on the % of energy

	**Sex-specific quartiles of the 5-CNS DI based on the % of energy**					
	**Q1 (best diet quality)**	**Q2**	**Q3**	**Q4 (worse diet quality)**	**P-trend**	**Per 1-unit increment**
Interquartile range (% of energy)	1.44-1.74	1.93-2.06	2.20-2.32	2.51-2.87		
Cases/n	35/157	41/158	51/157	44/156		
Model 4, OR (95% CI)	1 (Ref.)	1.56 (0.83-2.92)	2.40 (1.26-4.57)	2.06 (0.98-4.36)	0.026	1.71 (0.95-3.07)
Model 5, OR (95% CI)	1 (Ref.)	1.31 (0.70-2.44)	2.04 (1.06-3.92)	1.80 (0.81-3.97)	0.070	1.58 (0.84-2.97)

Continuous Nutri-Score DI based on the % of energy

	**Sex-specific quartiles of the Continuous Nutri-Score DI based on the % of energy**					
	**Q1 (best diet quality)**	**Q2**	**Q3**	**Q4 (worse diet quality)**	**P-trend**	**Per 1-unit increment**
Interquartile range (% of energy)	1.58-2.74	3.66-4.367	5.08-5.84	6.95-8.95		
Cases/n	31/160	45/157	46/157	49/153		
Model 4, OR (95% CI)	1 (Ref.)	1.62 (0.87-3.04)	2.33 (1.22-4.48)	2.24 (1.07-4.69)	0.020	1.06 (0.95-1.17)
Model 5, OR (95% CI)	1 (Ref.)	1.65 (0.88-3.09)	1.96 (1.01-3.80)	2.02 (0.93-4.37)	0.079	1.03 (0.92-1.15)

5-CNS**:** 5-Color Nutri-Score, DI: dietary index, OR: Odds Ratio, CI: Confidence Intervals.

Model 4 was adjusted as in Model 3 [for sex, age (continuous), total energy intake (kcal/d) and educational level (primary or less, secondary, and university), smoking (current, former, and never smoker former drinker status (yes/no), physical activity at leisure time (METS/h/week), time watching TV (h/week), total sleep time (minutes/day), total ethanol consumption (grams/day), body mass index (continuous), chronic obstructive pulmonary disease/asthma (yes/no), coronary heart disease (yes/no), hypercholesterolemia (yes/no), hypertension (yes/no), diabetes (yes/no), cancer (yes/no), arthrosis (yes/no), arthritis (yes/no), number of medications (0, 1 to 3, and >3), the MEDAS Index Score excluding wine (0-13 points)] with further adjustment for waist circumference at baseline (cm); and **Model 5** was adjusted as in model 3 with further adjustment for ultra-processed food consumption (sex-specific quartiles).
